# Supplementary material for: Siamese Networks for Clinically Relevant Bacteria Classification Based on Raman Spectroscopy
Source: Molecules. 2024 Feb 28;29(5):1061. doi: 10.3390/molecules29051061 (PMC10934697; doi:10.3390/molecules29051061)
Supplement: Supplementary file 1 [file molecules-29-01061-s001.zip › molecules-2832523-supplementary.pdf]

Supplementary material

Siamese Networks for Clinically Relevant Bacteria Classification based on Raman Spectroscopy

Jhonatan Contreras[1,2,‡], *Sara Mostafapour*[1,‡], *Jürgen Popp*[1,2] *and Thomas Bocklitz*[1,2,3,*]

[1] Institute of Physical Chemistry (IPC) and Abbe Center of Photonics (ACP), Friedrich Schiller University Jena, Member of the Leibniz Centre for Photonics in Infection Research (LPI), Helmholtzweg 4, 07743 Jena, Germany.

[2] Leibniz Institute of Photonic Technology, Member of Leibniz Health Technologies, Member of the Leibniz. Centre for Photonics in Infection Research (LPI), Albert Einstein Straße 9, 07745 Jena, Germany.

[3] Institute of Computer Science, Faculty of Mathematics, Physics & Computer Science, University Bayreuth Universitaetsstraße 30, 95447 Bayreuth, Germany

KEYWORDS: Siamese Networks; Machine learning; Bacteria Classification; Raman Spectroscopy.

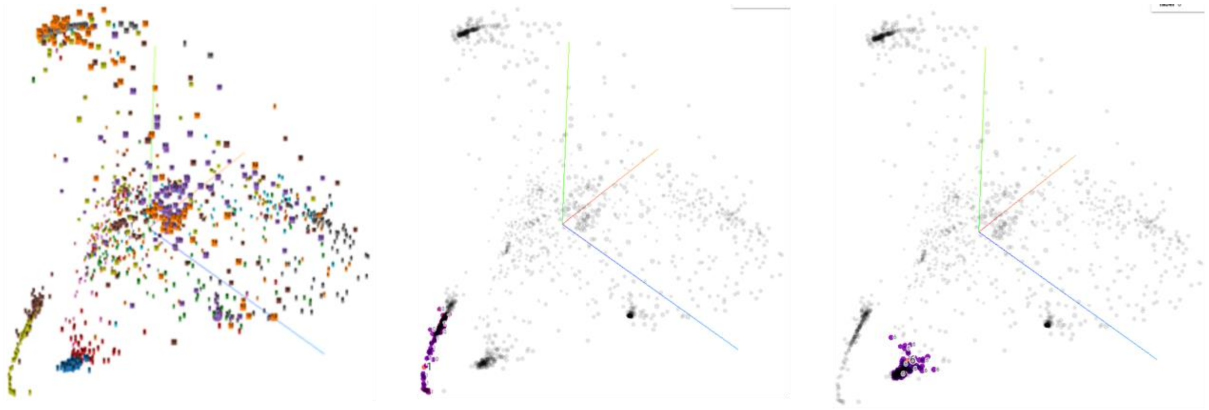

(a) (b) (c)

Figure S1. Pre-trained Embedding projections of three PCA components that capture only 52% of the variance in the testing data. (a) 15 classes clusters, (b)-(c) some of the clusters that are well separated from the other classes.

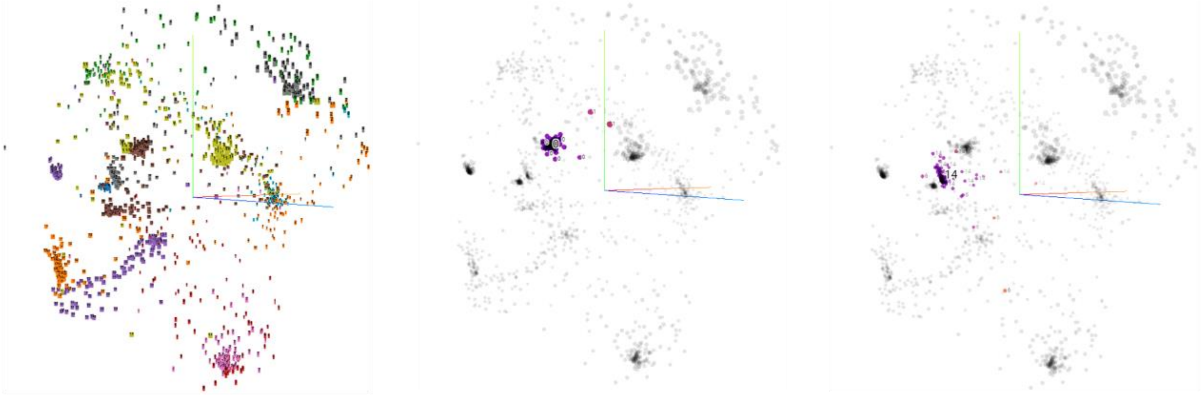

(a) (b) (c)

Figure S2. Fine-tuned Embedding projections of three PCA components that capture only 47% of the variance in the testing data. (a) 15 classes clusters, (b)-(c) some of the clusters that are well separated from the other classes.

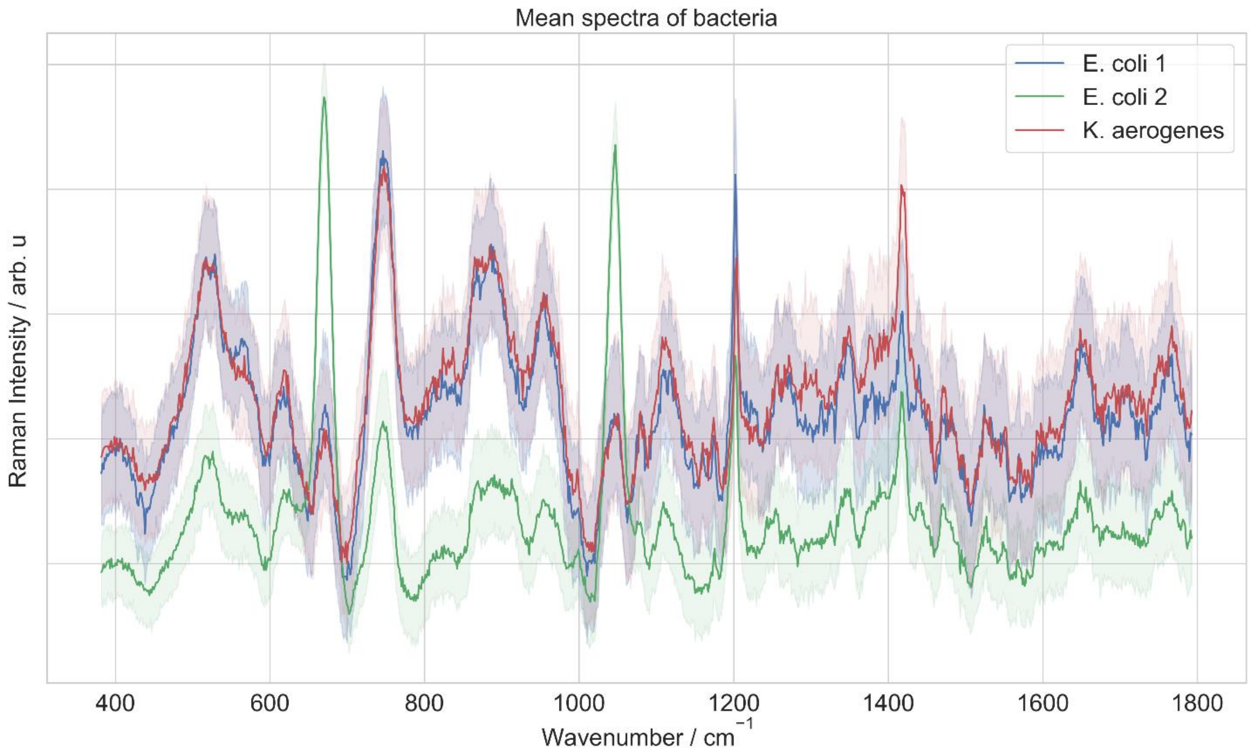

Figure S3. Mean spectra and standard deviation from training data for three bacterial strains.

Table S1. The species name, figure label, isolate code, and empiric antibiotic treatment. Data sourced from "Rapid identification of pathogenic bacteria using spectroscopy and deep learning", HO, Chi-Sing, et al. Nature communications, 2019.

Species	Figure label	Isolate code	Empiric antibiotic treatment
Escherichia coli	E. coli 1	ATCC 25922	Meropenem
Escherichia coli	E. coli 2	ATCC 700728	Meropenem
Klebsiella pneumoniae	K. pneumoniae 1	ATCC 33495	Meropenem
Klebsiella pneumoniae	K. pneumoniae 2	Stanford Clinical Collection	Meropenem
Klebsiella aerogenes	K. aerogenes	ATCC 13048	Meropenem
Enterobacter cloacae	E. cloacae	ATCC 13047	Meropenem
Proteus mirabilis	P. mirabilis	ATCC 43071	Meropenem
Serratia marcescens	S. marcescens	ATCC 13880	Meropenem
Pseudomonas aeruginosa	P. aeruginosa 1	ATCC 27853	Meropenem
Pseudomonas aeruginosa	P. aeruginosa 2	ATCC 9027	Meropenem
Staphylococcus aureus	MSSA 1	ATCC 25923	Vancomycin
Staphylococcus aureus	MSSA 2	ATCC 6538	Vancomycin
Staphylococcus aureus	MSSA 3	ATCC 29213	Vancomycin
Staphylococcus epidermidis	S. epidermidis	ATCC 12228	Vancomycin
Staphylococcus lugdunensis	S. lugdunensis	ATCC 49576	Vancomycin
Staphylococcus aureus	isogenic MSSA USA300-ex		Vancomycin
Staphylococcus aureus	MRSA 1 (isogenic)	USA300-wt	Vancomycin
Staphylococcus aureus	MRSA 2	ATCC 43300	Vancomycin

Streptococcus pneumoniae	S. pneumoniae 1	ATCC 49619	Ceftriaxone
Streptococcus pneumoniae	S. pneumoniae 2	ATCC 6305	Ceftriaxone
Streptococcus pyogenes (Group A)	Group A Strep.	ATCC 19615	Penicillin
Streptococcus agalactiae (Group B)	Group B Strep.	ATCC 12386	Penicillin
Streptococcus dysgalactiae (Group C)	Group C Strep.	ATCC 12388	Penicillin
Streptococcus dysgalactiae (Group G)	Group G Strep.	ATCC 12394	Penicillin
Streptococcus sanguinis	S. sanguinis	ATCC 35571	Penicillin
Enterococcus faecalis	E. faecalis 1	ATCC 29212	Penicillin
Enterococcus faecalis	E. faecalis 2	ATCC 51299	Penicillin
Enterococcus faecium	E. faecium	ATCC 700221	Daptomycin
Salmonella enterica	S. enterica	ATCC 13314	Ciprofloxacin
Candida albicans	C. albicans	ATCC 10231	Caspofungin
Candida glabrata	C. glabrata	ATCC 66032	Caspofungin

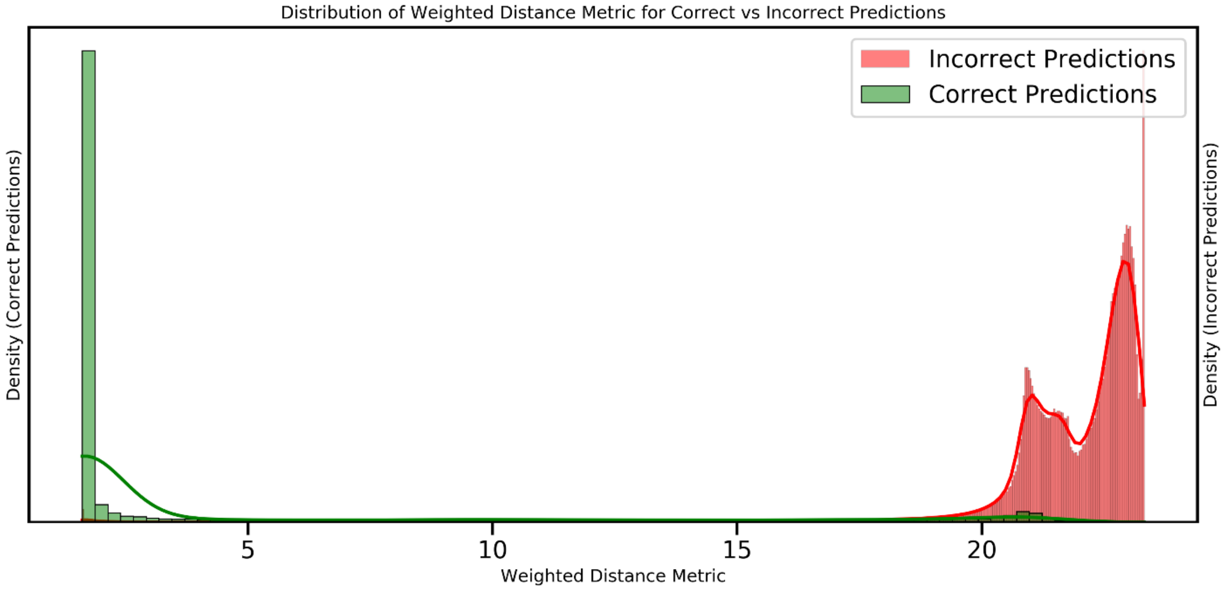

Figure S4. Distribution of Weighted Distance Metric for Correct vs Incorrect Predictions. Testing Data Across the 30 Bacteria Strains.

Table S2. Confusion matrix of different classification methods for six bacteria species dataset.

	PCA-LDA classification					
True\ Predicted	[1]	[2]	[3]	[4]	[5]	[6]
E. coli [1]	4356	100	72	2624	0	0
L. innocua [2]	76	6313	0	48	28	863
P. stutzeri [3]	585	1	6615	15	0	0
R. terrigena [4]	2260	452	3	4565	0	0
S. cohnii [5]	12	223	7	212	6117	613
S. warneri [6]	19	61	6	165	271	6678

	PLS-DA classification					
True\ Predicted	[1]	[2]	[3]	[4]	[5]	[6]
E. coli [1]	4466	130	128	2424	4	0
L. innocua [2]	158	6220	5	90	158	697
P. stutzeri [3]	414	4	6752	46	0	0
R. terrigena [4]	1832	691	5	4752	0	0
S. cohnii [5]	44	258	4	259	5721	898
S. warneri [6]	7	402	16	153	448	6174

	PCA-SVM classification					
True\ Predicted	[1]	[2]	[3]	[4]	[5]	[6]
E. coli [1]	4471	32	74	2327	225	23
L. innocua [2]	56	6330	16	46	64	816
P. stutzeri [3]	244	11	6934	10	0	17
R. terrigena [4]	1738	652	8	4837	41	4
S. cohnii [5]	50	297	8	53	6302	474
S. warneri [6]	24	766	11	45	258	6096

	PCA-RF classification					
True\ Predicted	[1]	[2]	[3]	[4]	[5]	[6]
E. coli [1]	4634	74	223	2202	15	4
L. innocua [2]	80	6186	31	125	142	764
P. stutzeri [3]	391	13	6793	15	4	0
R. terrigena [4]	2222	801	23	4211	13	10
S. cohnii [5]	51	261	19	137	6340	376
S. warneri [6]	30	417	1	118	489	6145

	Shallow CNN classification					
True\ Predicted	[1]	[2]	[3]	[4]	[5]	[6]
E. coli [1]	5030	58	72	1981	10	1
L. innocua [2]	45	6306	26	55	131	765
P. stutzeri [3]	231	21	6940	20	2	2
R. terrigena [4]	1623	498	47	5073	36	3
S. cohnii [5]	55	247	26	95	6452	309
S. warneri [6]	24	757	12	88	302	6017

	Deeper CNN classification					
True\ Predicted	[1]	[2]	[3]	[4]	[5]	[6]
E. coli [1]	5315	57	68	1642	49	21
L. innocua [2]	92	6262	11	65	105	793
P. stutzeri [3]	166	12	7030	6	2	0
R. terrigena [4]	1903	375	12	4887	90	13
S. cohnii [5]	46	141	11	101	6573	312
S. warneri [6]	14	273	1	79	249	6584

	Siamese model 2 classification					
True\ Predicted	[1]	[2]	[3]	[4]	[5]	[6]
E. coli [1]	4892	81	63	2092	5	19
L. innocua [2]	44	6343	20	69	119	733
P. stutzeri [3]	303	45	6855	13	0	0
R. terrigena [4]	1518	441	13	5297	11	0
S. cohnii [5]	12	334	9	114	6418	297
S. warneri [6]	0	477	0	59	210	6454
